# Treatment Response Prediction and Individualized Identification of Short-Term Abstinence Methamphetamine Dependence Using Brain Graph Metrics

**DOI:** 10.3389/fpsyt.2021.583950

**Published:** 2021-03-03

**Authors:** Cui Yan, Xuefei Yang, Ru Yang, Wenhan Yang, Jing Luo, Fei Tang, Sihong Huang, Jun Liu

**Affiliations:** ^1^Department of Radiology, Second Xiangya Hospital of Central South University, Changsha, China; ^2^The Key Laboratory of Biomedical Information Engineering, Ministry of Education, Department of Biomedical Engineering, School of Life Science and Technology, Xi'an Jiaotong University, Xi'an, China

**Keywords:** methamphetamine dependence, support vector machine, classification, treatment response, graph metrics

## Abstract

**Background:** The abuse of methamphetamine (MA) worldwide has gained international attention as the most rapidly growing illicit drug problem. The classification and treatment response prediction of MA addicts are thereby paramount, in order for effective treatments to be more targeted to individuals. However, there has been limited progress.

**Methods:** In the present study, 43 MA-dependent participants and 38 age- and gender-matched healthy controls were enrolled, and their resting-state functional magnetic resonance imaging data were collected. MA-dependent participants who showed 50% reduction in craving were defined as responders to treatment. The present study used the machine learning method, which is a support vector machine (SVM), to detect the most relevant features for discriminating and predicting the treatment response for MA-dependent participants based on the features extracted from the functional graph metrics.

**Results:** A classifier was able to differentiate MA-dependent subjects from normal controls, with a cross-validated prediction accuracy, sensitivity, and specificity of 73.2% [95% confidence interval (CI) = 71.23–74.17%), 66.05% (95% CI = 63.06–69.04%), and 80.35% (95% CI = 77.77–82.93%), respectively, at the individual level. The most accurate combination of classifier features included the nodal efficiency in the right middle temporal gyrus and the community index in the left precentral gyrus and cuneus. Between these two, the community index in the left precentral gyrus had the highest importance. In addition, the classification performance of the other classifier used to predict the treatment response of MA-dependent subjects had an accuracy, sensitivity, and specificity of 71.2% (95% CI = 69.28–73.12%), 86.75% (95% CI = 84.48–88.92%), and 55.65% (95% CI = 52.61–58.79%), respectively, at the individual level. Furthermore, the most accurate combination of classifier features included the nodal clustering coefficient in the right orbital part of the superior frontal gyrus, the nodal local efficiency in the right orbital part of the superior frontal gyrus, and the right triangular part of the inferior frontal gyrus and right temporal pole of middle temporal gyrus. Among these, the nodal local efficiency in the right temporal pole of the middle temporal gyrus had the highest feature importance.

**Conclusion:** The present study identified the most relevant features of MA addiction and treatment based on SVMs and the features extracted from the graph metrics and provided possible biomarkers to differentiate and predict the treatment response for MA-dependent patients. The brain regions involved in the best combinations should be given close attention during the treatment of MA.

## Introduction

Abuse of the synthetic psychostimulant, methamphetamine (MA), has gained international attention as the most rapidly growing illicit drug problem worldwide (1). In the 2017 World Drug Report, ~37 million people have become addicted to MA. MA abuse is usually accompanied by various health consequences, such as depression, anxiety, psychosis, cardiovascular disease, and human immunodeficiency virus, hepatitis B virus, hepatitis C virus that are spread by blood and sex behaviors due to the social environmental factor of MA use. Besides, a variety of secondary social costs were found, such as disruptions to family, school and work life, and violent behavior and drug-related crime (2, 3). Furthermore, frequent MA use can alter the release and activity of monoaminergic neurotransmitters, dopamine, norepinephrine, and serotonin and other unknown mechanisms on the central nervous system (CNS), which can be called neurotoxicity (4). The MA-induced harm on CNS further results in significant psychiatric withdrawal symptoms. Typical psychiatric symptoms include persecutory delusions, auditory hallucinations, and loss of insight that difficultly distinguish from some mental illnesses such as schizophrenia, and MA-induced withdrawal symptoms usually include anhedonia, hypersomnia, irritability, anxiety, aggression, and intense cravings for MA (5, 6). In general, the severity of these clinic symptoms depends on the amount of MA consumed.

MA abuse is termed as brain-based diseases given the neural mechanisms and clinical performance of MA use (7–9), which imply that the brain should contain information about an individual's current disease status and prognosis (10). At present, the diagnosis of drug use disorders mainly relies on descriptive signs and symptoms, according to the fourth or fifth edition of the *Diagnostic and Statistical Manual of Mental Disorders* (*DSM-IV* or *DSM-5*) criteria, in which a single “methamphetamine use disorder” with an added severity specification was listed (11). However, MA addicts, especially those with less severe symptoms, may deliberately mislead about the diagnosis because of their resistance to treatment. Therefore, an objective method to help diagnose MA addiction at the individual level is worth exploring. On the other hand, although there are many treatments available currently, few yielded convincing results of MA dependence (12, 13). At present, treatments of MA abuse are mainly based on two types: psychosocial treatments and pharmacologic treatments. Commonly used psychosocial treatments include cognitive behavioral therapy (14), contingency management (15, 16), motivational interviewing (17), and so on. Meanwhile, researchers explored many medications of MA treatment that frequently target dopaminergic, serotonergic, GABAergic, and/or glutamatergic brain pathways such as dexamphetamine (18, 19), methylphenidate (20, 21), naltrexone (22, 23), topiramate (24, 25), and so on. Therefore, it makes sense to predict the therapeutic effect before treatment, in order to allow doctors to identify the optimal treatment plan at the individual level. In this study, we defined MA-dependent participants with 50% reduction in craving as responders to treatment.

MA abuse was found to be associated with structural and functional alterations of the brain. In a study that used functional magnetic resonance imaging (fMRI), Zhang et al. researched the resting-state functional connectivity (RSFC) between seeds [region of interest (ROI)] selected with a higher value of ReHo and the whole-brain voxels and found different RSFC performance when compared with healthy controls (HCs) (26). Furthermore, in the studies conducted by Kohno et al. (27, 28), the midbrain and dorsolateral prefrontal cortex were chosen as ROIs, referring to previous research results. Kohno et al. reported that MA addicts differ from HCs in many ROI-related RSFCs, such as RSFC between the midbrain and caudate. These studies selected different ROIs based on different research aims, eventually presenting different results. Hence, RSFC based on ROI selection could not reveal the full spatial patterns of brain changes, and more suitable methods for determining differences in neural network activity are needed. Graph–theory–based complex network analysis is a non-invasive MRI-based tool that provides a powerful framework for examining the topological properties of brain networks and with no need for ROI selection. With this method, the nodes represent the brain regions, and the edges represent the anatomical or functional connections (FCs) among brain regions (29–32). The present study comprehensively examined the graph theoretical properties of the network structure after MA abuse, because it is unclear which graph metric is the most neurologically informative.

Equipped with machine learning techniques, researchers could make progress in identifying and predicting the treatment response for patients at the individual level. The support vector machine (SVM) is a multivariate pattern classification algorithm based on machine learning, and this could iteratively improve the performance in uncovering the relationships between variables by training classifiers (33). SVMs determine the hyperplane to separate multivariate features of two classes, allowing samples to be well-divided into two groups (such as patients and control groups). In studies that applied SVMs to identify MA-dependent patients, researchers have used features, including arterial spin labeling (34), task state fMRI data (35), heart rate extracted from MA-induced electrocardiogram (36), and differentially expressed genes (37). Compared to these methods, the present study selects features from various graph metrics. These graph metrics described the characteristics of brain networks from various aspects and comprehensively captured the functional information of the brain.

The present study aims to develop possible biomarkers for MA diagnosis and treatment prediction on an individual level, so that it provides a deeper understanding of the addiction and treatment of MA from the perspective of diagnosis and treatment prediction based on the graph–theory analysis and SVMs. The results of the present study represent an important step toward the development of traditional tools in imaging diagnostic identification, as well as the personalized treatment approaches for MA abstinence treatment in the future.

## Materials and Methods

The present study was carried out in accordance with the Drug Clinical Trial Management Regulations and approved by the Ethics Committee of the Second Xiangya Hospital of Central South University, which make decisions based on the Helsinki Declaration and International Ethical Guidelines for Biomedical Research Involving Human Subjects. An informed consent was obtained from each patient.

### Patient Population

In the present study, 43 MA-dependent participants (31.81 ± 8.50 years old, 28 males) and 38 age- and gender-matched HCs (35.21 ± 7.84 years old, 25 males) were recruited. The MA participants were collected from three drug rehabilitation centers in Hunan Province. In China, drug addicts are forced to receive treatment in the drug rehabilitation center for at least 2 years. During this period, they have no access to any drugs. In our study, MA participants were also forced to serve a 2-year sentence at the rehabilitation center. All MA patients volunteered to participate in the experiment and were compensated for their time with 1-month reduction of their sentence. All subjects reported a detailed drug use history during the face-to-face interview. MA addicts were included with positive urine test for MA, negative urine test for other drugs, and the diagnosis of addiction based on the *DSM-IV*. Subjects who had comorbid or history of psychiatric illness (e.g., schizophrenia and major depression unrelated to drug withdrawal), neurological disorder (e.g., multiple sclerosis, Parkinson disease, other primary degenerative brain diseases, and any brain infections or neoplasms), head trauma, or major chronic medical illnesses (e.g., diabetes, uncontrolled hypertension, and heart disease) were excluded. In addition, subjects with pregnant or contraindications to MRI (e.g., metallic, electronic devices, or implants) were excluded. Patients who met the aforementioned criteria were involved in the present study. HCs were recruited through WeChat, QQ, and poster with the same inclusion and exclusion criteria applied for MA users.

Information on the demographics was gathered before the brain imaging was performed (e.g., age, gender and educational attainment). Furthermore, the Fagerström Test for Nicotine Dependence (FTND) and the Alcohol Use Disorder Identification Test (AUDIT) were collected to evaluate for cigarette smoking and alcohol use. The severity of present MA craving was measured before the fMRI, with the Methamphetamine Craving Questionnaire (MCQ) adapted from the Heroin Craving Questionnaire.

All MA subjects underwent two examinations. The first examination was performed from 1 week to 1 month after abstinence (MA1 group), and the second examination was performed at ~1.5 years after abstinence (MA2 group). During this abstinence period, MA-dependent participants had no access to anything related to MA and received different types of drug treatment, including only traditional Chinese medicine (*n* = 12), only antidepressants and antianxiety medications (*n* = 10), both of these (*n* = 19), and placebo (*n* = 2) for 2 months after the first MRI examination. Before each MRI examination, these patients completed the MCQ.

### MRI Data Acquisition and Preprocessing

All MRI data were acquired using a 3-T Siemens Skyra MRI scanner (Magnetom Skyra, Siemens, Germany) equipped with a 32-channel head coil. The structural T1-weighted three-dimensional magnetization-prepared rapid acquisition with gradient echo was acquired with the following parameters: 176 sagittal slices, slice thickness = 1 mm, gap = 0 mm, field of view (FOV) = 256 × 256 mm, repetition time (TR) = 1,450 ms, echo time (TE) = 2.03 ms, inversion time (TI) = 900 ms, flip angle = 30°, and voxel size = 1 × 1 × 1 mm^3^.

Resting-state functional MRI scans were acquired using the following parameters: 36 axial slices, thickness = 4 mm, FOV = 220 × 220 mm, TR = 2,000 ms, TE = 30 ms, flip angle = 80°, and 225 volumes. All subjects were placed in the supine position with eyes open and foam padding between their head and the head coil to minimize head motion.

The resting-state fMRI data were preprocessed using the DPABI (version 3.0, http://rfmri.org/dpabi) software, which include the slice timing, realignment, spatial normalization, regression, smoothing, and filter. For functional image preprocessing, the first 10 volumes were discarded to avoid signal instability. Slice timing was conducted to correct the remaining volumes, and head realign was used to reject abnormal subjects who had more than 2 mm of motion or 2.0° of rotation. The realigned images were transformed to the Montreal Neurological Institute standard space using the DARTEL algorithm. Then, the signals were regressed out, including the global signal, cerebrospinal fluid signal and white matter signal, and the 24 head motion parameters. Afterward, the images were smoothed with the Gaussian kernel using the full width at half maximum of 6 mm and bandpass temporal filtered (0.01–0.1 Hz) to reduce the low-frequency drift and physiological high-frequency noise, including the breath and heartbeats (26).

### Calculation of Graph Metrics

A total of 15 graph metrics were calculated using the GRETNA software (version 2.0.0, https://www.nitrc.org/projects/gretna/), which included seven global network metrics: clustering coefficient, shortest path length, global efficiency, local efficiency, assortativity, synchronization, and hierarchy, and eight nodal network metrics: nodal clustering coefficient, nodal shortest path length, nodal efficiency, nodal local efficiency, degree centrality, betweenness centrality, community index, and participant coefficient. The Anatomical Automatic Labeling template was used to extract the average time series of the blood oxygen level–dependent (BOLD) signals in each brain region. There were 90 regions across the whole brain. Next, the correlation between each pair of the regional time series of the BOLD signal was calculated as FCs, resulting in 4,005 (90 × 89/2) FC values, excluding the self-correlation. Then, these FCs were used to calculate the graph metrics for each subject, with a sparsity threshold ranging from 0.05 to 0.50, in steps of 0.05. For features extracted from graph metrics, each subject has one value with the whole brain for global network metrics and 90 values with 90 brain regions for nodal network metrics. Afterward, these features were reduced into the top features in dimension that could be entered into the SVMs also with the same sparsity threshold.

### Statistical Analysis

The statistical analysis was performed on the graph metrics (7 × 1 + 8 × 90, 727 features) to extract the top features using the GRETNA software. General linear model was performed between the MA1 group and HC group, with education level, FTND, and AUDIT as the covariables. Repeated-measures analysis of variance (ANOVA) was performed between the MA1 group and MA2 group for each graph metric. A strict false discovery rate of 0.05 was applied to the analysis results as the multiple-comparisons correction. Then, features that significantly differed between groups were considered as the top features and entered into the SVMs. The top features that differed between the MA1 group and HC group were extracted from these two groups and entered into the classifier for identifying those with MA addiction. In addition, another classifier for predicting the treatment response received top features extracted from the MA1 group, which differed between the MA1 group and MA2 group.

### SVM-Based Classification

Two classifiers were conducted with one for investigating the best top feature combination to differentiate patients from controls and the other for investigating the best top feature combination to predict the treatment response of MA addicts. This was performed using the SVMs implemented in the LIBSVM classification library (38, 39). The adopted linear kernel allowed for the interpretation of the weight vector (i.e., the relative importance of each feature in the classifier). Then, the weight vectors were subsequently used to rank the importance of the features in identifying MA. The SVMs were initially trained using all top features derived from the graph metrics. In order to identify the most informative feature, the least important feature (in terms of weight vector) was removed after each round of SVM training, and a new SVM was trained with the remaining features. This process was repeated until only a single feature remained. The accuracy of the classifier was recorded at each stepwise removal. Leave-one-out cross-validation was used, in which one subject was iteratively left out as the testing target, and the remaining samples were used to train the SVMs. Each SVM was repeated for 1,000 times (40, 41).

A patient was termed as a responder if the MCQ scores exhibited a 50% decrease or more. The percentage drop of the MCQ score was calculated, as follows:

(1) $$\begin{array}{l} d=\frac{{\text{MCQ}}_{\text{first}}{\text{-MCQ}}_{\text{second}}}{{\text{MCQ}}_{\text{first}}}. \end{array}$$

According to the value of *d*, the MA-dependent participants were divided into two groups: responder group and non-responder group. Then, the SVM was used to classify whether the MA-dependent participant would be a responder after the abstinence treatment.

## Results

### Patient Population

In the process of image preprocessing, nine MA-dependent participants and three HCs could not survive from the realignment, ultimately resulting in the involvement of 34 MA-dependent participants and 35 HCs in the present study. The demographic characteristics of the MA and HC groups are presented in Table 1. Among the 34 MA-dependent participants, there were 21 responders and 13 non-responders, and their demographic characteristics are presented in Table 2.

**Table 1:** Clinical characteristics of the participants (mean ± SD or median [IQR]).

**Characteristics**	**MA1 (*n* = 34)**	**MA2 (*n* = 34)**	**HC (*n* = 35)**	***T* value**	***P*-value**
Age (years)	32.62 ± 8.80	—	35.14 ± 7.94	−1.25	0.22
% Male	58.8	—	62.9	0.12	0.81
Education level (years)	8.87 ± 2.49	—	11.19 ± 2.78	−3.65	0.001
Duration of MA use (years)	4 (2.88,6.75)	—	—	—	—
Average abstinence (days)	21.5 (8.75,45.25)	406.50 (331.75,447.50)	—	5.09	<0.001
FTND	4.74 ± 2.29	—	2.34 ± 2.55	−4.10	<0.001
AUDIT	3.5 (0,8)	—	0 (0,3)	−2.62	0.009
MCQ	55.50 (5.50,61.00)	25.50 (0,32.25)	—	−4.78	<0.001

**Table 2:** Demographic characteristics of the responders and non-responders of MA-dependent participants [mean ± SD or median (IQR)].

**Characteristics**	**Responders (*n* = 21)**	**Non-responders (*n* = 13)**	***P*-value**
Age (years)	30.81 ± 8.27	35.54 ± 9.15	0.13
% Male	92.31%	61.9%	0.002
Education level (years)	9.28 ± 1.74	8.19 ± 3.35	0.22
FTND	4.71 ± 2.41	4.76 ± 2.16	0.95
AUDIT	4 (0,8)	3 (0,9)	0.85

### Classification Performance of MA-Dependent Patients

**Figure 1:**
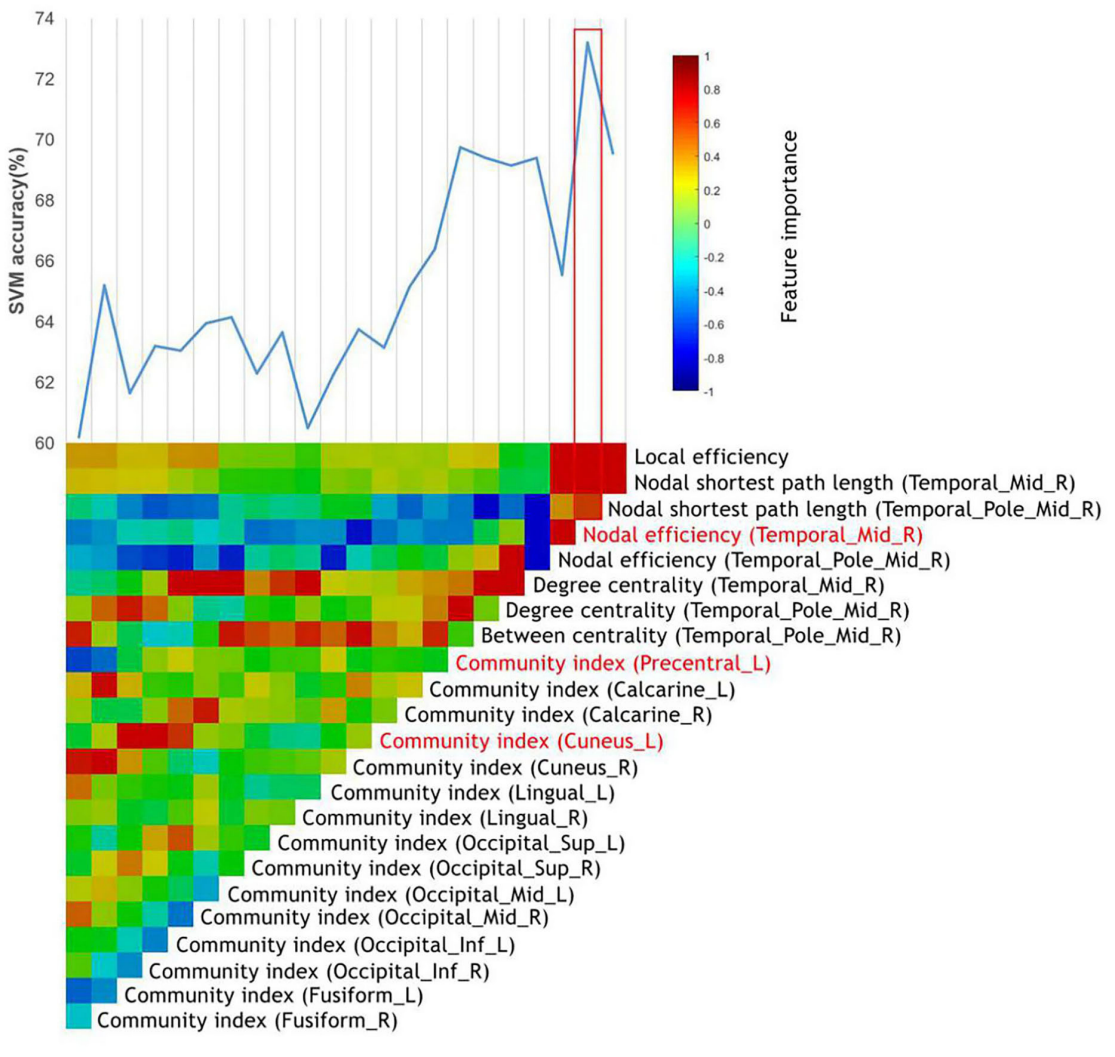
The identification of MA-dependent patients using the graph metrics. The vertical color bar represents the relative feature importance of all top features when used together in training the SVM to differentiate MA-dependent patients. The stepwise removal of the least influential feature was repeated until a single top feature remained. At each stage of the feature removal, the accuracy of the retrained SVMs was recorded above the color bars. The most influential feature combination was outlined in red, which included the top features highlighted in red.

After the general linear model was performed, 23 top features remained, and all these top features were entered into the SVMs. Through the stepwise removal of the top features, the investigators were able to arrive at an optimal combination, which identified patients in 73.2% [95% confidence interval (CI) = 71.23–74.17%] of the cases (Table 3A). The progressive removal of the first 20 top features (in order of increasing feature importance) increased the classification accuracy, and this was the result of the reduced overfitting in SVMs. The most accurate combination of classifier features included the nodal efficiency in the right middle temporal gyrus (MTG.R), and the community index in the left precentral gyrus (PreCG.L) and cuneus (CUN.L), in which the community index in PreCG.L had the highest importance. The SVM accuracy decreased when additional top features were added or subtracted from the optimal combination. The accuracy and evolved top features of each SVM are shown in Figure 1.

**Table 3A:** Individual identification of MA-dependent patients.

***N* patients (*N* HC)**	**Accuracy**	**Sensitivity**	**Specificity**
69 (35)	73.2% (95% CI: 71.23–74.17%)	66.05% (95% CI 63.06–69.04%)	80.35% (95% CI: 77.77–82.93%)

### Classification Performance of Treatment Response Prediction

**Figure 2:**
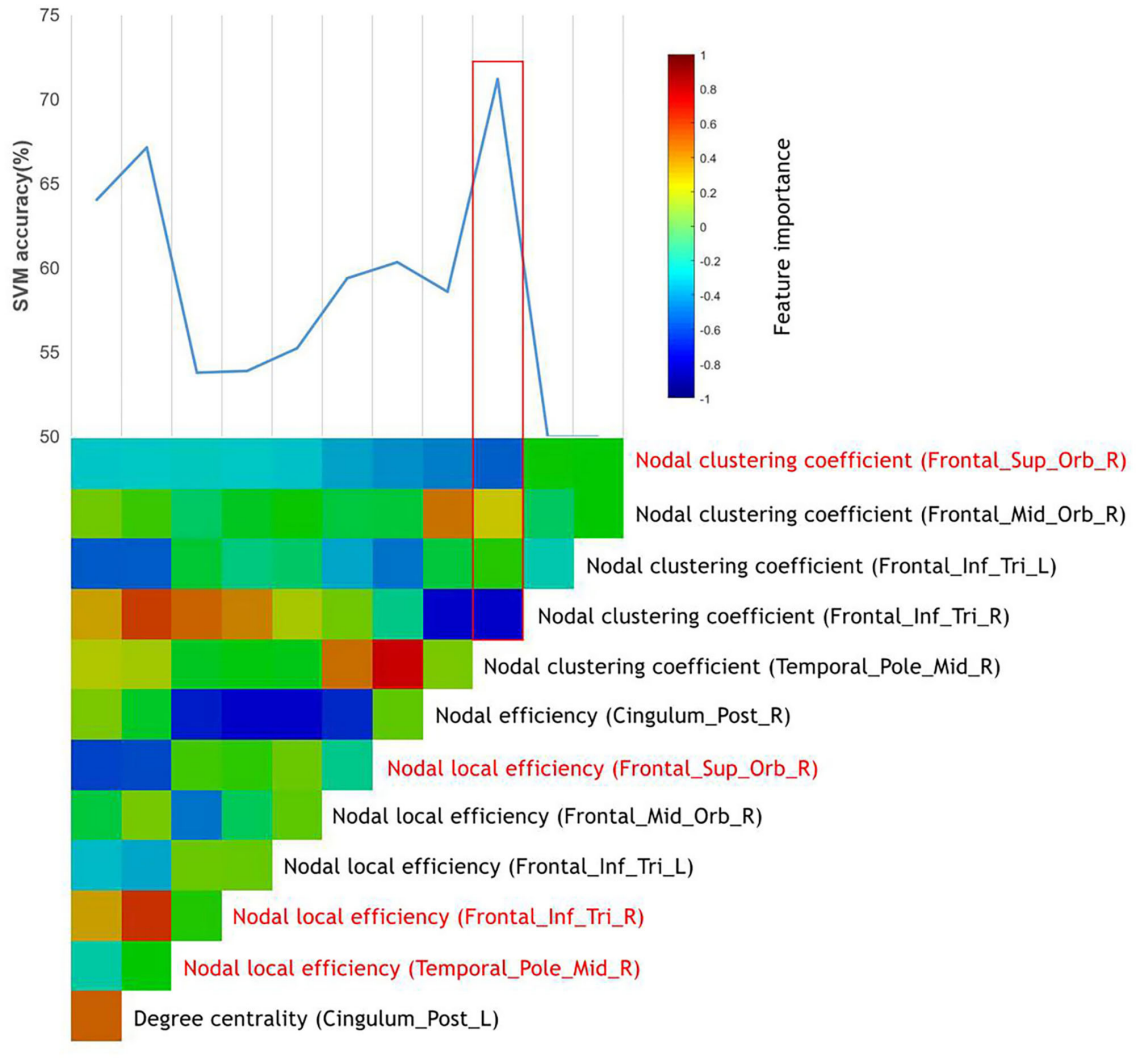
The prediction of responders of MA-dependent patients to abstinence treatment using graph metrics. The vertical color bar represents the relative feature importance of all top features when used together in training an SVM to differentiate the responders of MA-dependent patients. The stepwise removal of the least influential top feature was repeated until a single top feature remained. At each stage of the feature removal, the accuracy of the retrained SVMs was recorded above the color bars. The most influential feature combination was outlined in red, which included the top features highlighted in red.

After the ANOVA was performed, 12 top features remained, and all these top features were entered into the SVMs. Through the stepwise removal of the top features, the investigators were able to arrive at an optimal combination, which identified patients in 72.2% (95% CI = 69.28–73.12%) of the cases (Table 3B). The progressive removal of the first eight top features (in order of increasing feature importance) increased the classification accuracy. The most accurate combination of classifier features included the nodal clustering coefficient in the right orbital part of superior frontal gyrus (ORBsup.R), the nodal local efficiency in ORBsup.R, the right triangular pat of the inferior frontal gyrus (IFGtriang.R), and right temporal pole of the middle temporal gyrus (TPOmid.R). Among these, the nodal local efficiency in TPOmid.R had the highest feature importance. Figure 2 presents the accuracy and evolved top features of each SVM.

**Table 3B:** Individual prediction of responders to the abstinence treatment of MA-dependent patients.

***N* patients (*N* responders)**	**Accuracy**	**Sensitivity**	**Specificity**
34 (21)	72.2% (95% CI = 69.28–73.12%)	86.75% (95% CI = 84.48–88.92%)	55.65% (95% CI = 52.61–58.79%)

In order to further validate the classification performance (i.e., generalization) of the classifiers, these results were then replicated in a separate test group of patients including 10 MA-dependent patients (four responders and six non-responders) and 10 HCs. These features differentiated the independent test data with the accuracy, sensitivity, and specificity of 67.7% (95% CI = 65.7–69.69%), 61.7% (95% CI = 58.69–64.81%), and 73.65% (95% CI = 70.82–76.48%), respectively, and predict the responders with the accuracy, sensitivity, and specificity of 67.2% (95% CI = 65.40–69.40%), 58.2% (95% CI = 55.1–61.36%), and 76.2% (95% CI = 73.35–79.05%).

## Discussion

In the present study, the investigators filtered informative features about MA addiction and treatment by the training of classifiers using the SVM combined with the graph metrics, which could provide possible biomarkers to identify and predict the treatment response for MA-dependent patients at the individual level.

In the classifier for identifying MA-dependent patients, it was found that the combination of nodal efficiency (MTG.R), community index (PreCG.L), and community index (CUN.L) exhibits the best performance. In the graph theory, the nodal efficiency for a given node characterizes the efficiency of the parallel information transfer of that node in the network and quantifies the importance of this node for the communication within the network (42). In addition, the community index indicates which community or modularity a given node belongs to. Changes in the community index generally suggest the changes of locally connected clusters or modules implicated in specialized information processing (43). Both of these were used to describe the location and efficiency of nodes in the information transfer. As for the regions observed in present study, the middle temporal gyrus was previously described as the dorsal visual streams that encoded the spatial information among objects (44). A previous study also reported an activation in response to less complex visual images in the posterior right middle temporal gyrus when healthy adult participants received MA, and it was concluded that MA may change the relative sensitivity of higher-order sensory processing (45). The precentral gyrus is well-recognized as the primary motor cortex, and this was reported to have a reduced activation, when compared to HCs, in performing the emotion-matching task with fearful non-face images (46). The reduced gray matter of the left precentral gyrus was consistently observed in study of Hall et al. (47). In addition, Van Hedger et al. (48) reported the greater activation in the bilateral cuneus when exploring the neural responses correlated to the visual–auditory stimuli paired with MA in healthy people. These three brain regions were all involved in the sensory and emotion processing. The altered nodal efficiency or community index of these regions may affect the correction and efficiency of information translation and damage the integrity of sensory and emotional pathway. These abnormalities are partly correlated to the negative emotions and hallucinations of MA patients. Interestingly, these present results mainly revealed the sensory and emotion processing-related brain regions, instead of the dopaminergic reward circuits (49), providing support to the possibility that sensory effects could also become conditioned (50). Furthermore, it was speculated that the diagnosis of MA is more closely correlated to the sensory and emotion function and that the combination of features mentioned above could be a potential subjective predictable biomarker for MA abuse.

On the other hand, in the classifier for predicting the responders of MA-dependent patients, the investigators found that the combination of nodal clustering coefficient (ORBsup.R), nodal local efficiency (ORBsup.R), nodal local efficiency (IFGtriang.R), and nodal local efficiency (TPOmid.R) performed best. The nodal clustering coefficient of a given node describes the range or amount of potential connections that the closest neighbors of the node actually have and measures the likelihood that its neighborhoods are connected to each other (51, 52). In addition, the nodal local efficiency is the global efficiency computed on the neighborhood of the node and measures how efficient the communication is among the first neighbors of this node when it is removed (43). Both metrics describe the importance of nodes in brain networks, and changes of these, usually in mean disordered network functions. The activation of the orbitofrontal cortex and the temporal poles is correlated to empathy ability. Kim and colleagues (53) reported hypoactivation in the orbitofrontal cortex and temporal poles in MA abusers relative to healthy subjects during empathy progression. This may be correlated to the lower levels of dopamine D_2_ receptor availability and decreased dopamine transporter density in the orbitofrontal cortex and temporal poles in the MA abusers (54, 55). During the social interaction, empathy plays a pivotal role that enables the understanding of others' thoughts well and predicts their future actions, allowing for better social communication. Disordered empathy in MA abusers could reduce impairments in social interactions that contribute to stress, which may increase the negative mood states and the risk of relapse. In addition, Paulus et al. (56) suggested that the integrity of the orbitofrontal cortex is important for decision-making when the results are uncertain. Similarly, the inferior temporal cortex has been considered to be involved in the brain networks of decision-making (57). Using a two-choice prediction task with fixed error rates, Stewart et al. (58) reported the reduced activation in the inferior temporal cortex, which is suggestive of reduced attentional resources allocated to decision making. These provide insight into the nature that MA abusers, who usually ignore the risk factors of drug use, exhibit compulsive drug use behavior. Taken together, these present results suggest that better treatment may improve the impaired network function of the orbitofrontal cortex, temporal poles, and the inferior temporal cortex, rehabilitating patient's ability of empathy and decisions making, to some extent. Improved interpersonal communication could further improve the patient's negative mood status, making MA withdrawal smoother. The restoration of decision-making ability also enables patients to make choices that are better for their own health. The present study also provides a starting point for studying the recovery mechanism of brain regions damaged by MA and a new thought about therapeutic targets from the perspective of treatment prediction.

Several considerations need to be taken into account with respect to the interpretation of these present findings. First, the sample size of the present study was limited because of the inherent challenges in recruitment of MA dependents. Usually, the machine learning method needs a large sample size to obtain good classification results. Hence, the smaller sample size in the present study may have had an impact the classification accuracy. A larger sample replication would strengthen the generalizability of the present methods and allow for the full exploration of the parameter and feature space. Moreover, because of the limited sample size in different treatment groups, we were not able to distinguish between different treatment groups. Second, The MA and HC groups differed by not only MA use but also education, nicotine, and alcohol use. Although we have removed them as covariables when we performed general linear model between these two groups, it would be difficult to claim that the classifier is only differentiating based on MA use. Third, future studies may go beyond defining responders to treatment by those who showed 50% reduction in craving and, instead, use abstinence criteria. This was not possible in our study because of the strict abstinence policy of the rehabilitation centers from which we drew our participants. Finally, the individual identification of MA-dependent patients was merely based on a sample that included MA-dependent patients and HCs. Hence, patients should be collected in future studies who experience first-episode psychosis in association with conditions other than MA, such as heroin or cocaine.

## Conclusion

The present study identified the most relevant features of MA addiction and treatment based on SVMs and the features extracted from the graph metrics and provided possible biomarkers to differentiate and predict the treatment response for MA-dependent patients. Brain regions involved in the best combinations need to be given close attention during the treatment of MA. The present study provides a step toward the individualized identification and treatment response prediction of MA-dependent patients, laying a basis for precision medicine approaches.

## Data Availability Statement

The raw data supporting the conclusions of this article will be made available by the authors, without undue reservation.

## Ethics Statement

The studies involving human participants were reviewed and approved by the Ethics Committee of the Second Xiangya Hospital of Central South University. The patients/participants provided their written informed consent to participate in this study. Written informed consent was obtained from the individual(s) for the publication of any potentially identifiable images or data included in this article.

## Author Contributions

JLiu conceived of the study. RY contributed to study design and manuscript modification. CY and XY performed analysis and drafted the manuscript. WY, JLuo, and FT performed data collection. SH performed data processing and statistical analysis. All authors revised the manuscript and have read and approved the final version of the manuscript.

## Conflict of Interest

The authors declare that the research was conducted in the absence of any commercial or financial relationships that could be construed as a potential conflict of interest.
